# Analysis of Streamflow Complexity Based on Entropies in the Weihe River Basin, China

**DOI:** 10.3390/e22010038

**Published:** 2019-12-26

**Authors:** Weijie Ma, Yan Kang, Songbai Song

**Affiliations:** College of Water Resources and Architectural Engineering, Key Laboratory of Agricultural Soil and Water Engineering in Arid and Semiarid Areas, Ministry of Education, Northwest Agriculture and Forest University, Yangling 712100, China; 13007539880@163.com

**Keywords:** complexity, streamflow, approximate entropy, sample entropy, two-dimensional entropy, fuzzy entropy

## Abstract

The study on the complexity of streamflow has guiding significance for hydrologic simulation, hydrologic prediction, water resources planning and management. Utilizing monthly streamflow data from four hydrologic control stations in the mainstream of the Weihe River in China, the methods of approximate entropy, sample entropy, two-dimensional entropy and fuzzy entropy are introduced into hydrology research to investigate the spatial distribution and dynamic change in streamflow complexity. The results indicate that the complexity of the streamflow has spatial differences in the Weihe River watershed, exhibiting an increasing tendency along the Weihe mainstream, except at the Linjiacun station, which may be attributed to the elevated anthropogenic influence. Employing sliding entropies, the variation points of the streamflow time series at the Weijiabu station were identified in 1968, 1993 and 2003, and those at the Linjiacun station, Xianyang station and Huaxian station occurred in 1971, 1993 and 2003. In the verification of the above points, the minimum value of *t*-test is 3.7514, and that of Brown–Forsythe is 7.0307, far exceeding the significance level of 95%. Also, the cumulative anomaly can detect two variation points. The *t*-test, Brown–Forsythe test and cumulative anomaly test strengthen the conclusion regarding the availability of entropies for identifying the streamflow variability. The results lead us to conclude that four entropies have good application effects in the complexity analysis of the streamflow time series. Moreover, two-dimensional entropy and fuzzy entropy, which have been rarely used in hydrology research before, demonstrate better continuity and relative consistency, are more suitable for short and noisy hydrologic time series and more effectively identify the streamflow complexity. The results could be very useful in identifying variation points in the streamflow time series.

## 1. Introduction

Streamflow, as a key component of the hydrologic cycle, is significantly influenced, and its natural variability is being complicated by climate change and human activities, which may introduce extra chaotic information into discharge records [1]. It is characterized with a stochastic, nonlinear, non-stationary and time-varying nature by a multitude of uncertain information and represents a complex system [2,3,4]. Extreme floods and droughts are generally related to those aggravating variability of discharge, destroying the consistency of hydrologic data and complicating hydrologic research, such as hydrologic simulation and prediction [5]. It is vital to investigate the complexity of streamflow variability for effectively depicting the hydrologic process, planning and managing of water resources.

A variety of methods have been utilized to characterize the complexity of the system [6,7,8,9,10]. Some of the most common approaches include Lyapunov exponent, fractal dimension, Lempel–Ziv algorithm and Kolmogorov entropy. However, those classical methods are sensitive to noise, and to obtain credible results, they require a long time series, which is either unavailable for streamflow data or practically cumbersome to process if available [11]. The entropy algorithm, stemming from information theory, is considered an effective measure of uncertainty or randomness of a series of data without any previous knowledge about the source generating the dataset [12]. Information entropy and its derivative algorithms allow us to measure the amount of information in a mathematical way and have been extensively employed in multitude of research fields [13,14,15,16], including hydrology. It has been achieved in detecting the randomness and complexity of streamflow based on approximate entropy (ApEn) and sample entropy (SampEn) [11,15].

Approximate entropy, presented by Pincus [17], has shown the characteristics of strong anti-interference ability and requirement of a short length of data. However, ApEn is a biased statistic, with self-matches in calculating the similarity of vectors, resulting in a certain deviation for the final results [18,19]. As an improved algorithm of ApEn, sample entropy (SampEn) was proposed by Richman [20], which can eliminate the error caused by self-matches of vectors and has the advantages of better relative consistency, less dependence on data length, and faster operational speed compared with those of ApEn. ApEn and SampEn, with the same physical meaning, represent the level of self-similarity of patterns in the reconstructed *m*-dimensional phase space and the probability of generating new patterns when the dimension increases [21,22]. However, SampEn does not consider the influence on the time series complexity of the distribution of similar vectors and the complexity of the vectors composing the sequence.

In order to eliminate the bias, Yu [23] proposed a modified algorithm of SampEn called two-dimensional entropy (TD-entropy) and introduced the time distance between vectors and the mode distance into the vector similarity measurement for analysing the complexity of financial time series. In addition, the effect of the degree of patterns of self-similarity on the sequence complexity is taken into account in calculating the entropy value. The TD-entropy has better consistency [23] and continuity, and it may be more reasonable than SampEn for measuring the complexity of hydrologic sequences.

In the above entropies, the similarity of vectors is determined by the Heaviside function with rigid boundaries, whose hard threshold will lead us to obtain unstable results. Fuzzy entropy (FuzzyEn), as a new family of statistics, is developed by Chen [24], where the concept of Zadeh’s fuzzy sets are introduced [25] and the Heaviside function is replaced by the fuzzy membership function with soft and continuous boundaries, which improve the unstable results of the similarity of the vectors. Moreover, FuzzyEn features advantages of better continuity, relative consistency, free parameter selection and stronger robustness [26,27].

Among those four entropies, ApEn and SampEn have been introduced into the hydrologic system for detecting the dynamic changes and variation in the hydrologic series and have been validated [11,28,29]. TD-entropy and FuzzyEn have rarely been employed to identify the complexity of hydrologic series before. However, TD-entropy and FuzzyEn have been widely used for mechanical fault diagnosis [14,30], stock market analysis [23] and image segmentation [31], and the literatures [32,33] found that fuzzy entropy has good stability and reliability when used to study the complexity of precipitation. The researches have shown that fuzzy entropy equips the capabilities of better continuity and stronger anti-interference in detecting the complexity than those of ApEn and SampEn. Moreover, TD-entropy, taking into account the effect of self-similarity of patterns, measures the complexity from the perspective of probability corresponding to the randomness of the hydrologic series. The concept of fuzzy sets in FuzzyEn is consistent with the fuzziness of the hydrologic series. Therefore, the properties of TD-entropy and FuzzyEn make it more reasonable to analyse the complexity of hydrologic series. This study will attempt to introduce these two entropies into hydrology and to explore the effectiveness of methods in the complexity analysis of hydrologic series to provide effective methods for identifying streamflow complexity.

In this paper, entropies, including ApEn, SampEn, TD-entropy and FuzzyEn, are combined with sliding technique, are employed to investigate the streamflow complexity of the Weihe River in China for the period 1954–2016. The objectives of this paper are (1) to analyse the spatial difference of the streamflow complexity in the main stream of the Weihe River, based on data from Linjiacun station, Weijiabu station, Xianyang station and Huaxian station, using ApEn, SampEn, TD-entropy and FuzzyEn; (2) to evaluate the dynamic variability in streamflow complexity for four gauging stations by introducing the sliding technique into ApEn, SampEn, TD-entropy and FuzzyEn to diagnose the variation points of the streamflow series; and (3) to compare the four derived sliding-entropies and to verify the availability of sliding-entropies for identifying the complexity by employing the sliding *t*-test, Brown–Forsythe test and cumulative anomaly test. Subsequently, the important results and conclusions will be presented.

## 2. Methods

### 2.1. Approximate Entropy

Assuming an N-point time series {u(1),u(2),…,u(N)}, given the embedded dimension *m*, reconstruct a new set of vectors from the series {u(i)} as follows:

(1) $$X_{i}^{m}=\{u(i),u(i+1),\ldots ,u(i+m-1)\},\;i=\;1,2,\;\ldots ,\;N-\;m+\;1$$

Calculate the distance between $X_{i}^{m}$ and $X_{j}^{m}$ as follows:

(2) $$d_{i,j}^{m}=d\{X_{i}^{m},X_{j}^{m}\}=\max\{|u(i+k)-u(j+k)|\},\;k=\;0,\;1,\;\ldots ,\;m\;-\;1$$

which is equal to the maximum of the absolute difference between the corresponding elements of the vectors. Give the threshold value *r* (*r* = 0.1~0.25 SD, SD is the standard deviation of the original time series) [17]. For each *i* ≤ *N* – *m* + 1, calculate the number of vectors $X_{j}^{m}$ within *r* of $X_{i}^{m}$, denoted as $B_{i}$.

(3) $$B_{i}=\sum_{j=1}^{N-m+1}\{\begin{array}{c} 1,d_{i,j}^{m}\leq r\\ 0,d_{i,j}^{m}>r \end{array}$$

Construct

(4) $$C_{i}^{m}(r)=\frac{B_{i}}{N-m+1}$$

and

(5) $$C^{m}(r)={\left(N-m+1\right)}^{-1}\sum_{i=1}^{N-m+1}\ln C_{i}^{m}(r)$$

where ln is the natural logarithm. Increase the dimension to *m* + 1 and repeat the above steps to get $C_{i}^{m+1}(r)$ and $C^{m+1}(r)$. Thus, approximate entropy can be defined as $\underset{N\to \infty }{\lim}[C^{m}(r)-C^{m+1}(r)]$ and is usually estimated by [18] the following:

(6) $$ApEn(m,r,N)=C^{m}(r)-C^{m+1}(r)$$

### 2.2. Sample Entropy

For an *N*-point time series {u(1),u(2),…,u(N)}, give the phase space dimension *m* and form a sequence of vector $X_{1}^{m}$ through $X_{N-m+1}^{m}$. The detailed form is as follows:

(7) $$X_{i}^{m}=\{u(i),u(i+1),\ldots ,u(i+m-1)\},\;i=\;1,\;\ldots ,\;N\;-\;m+\;1$$

where *m* denotes each vector of length. Define the distance between $X_{i}^{m}$ and $X_{j}^{m}$ as follows:

(8) $$d_{i,j}^{m}=d\{X_{i}^{m},X_{j}^{m}\}=\max\{|u(i+k)-u(j+k)|\},\;k=\;0,\;1,\;\ldots ,\;m-1$$

Given a tolerance *r*, let $C_{i}^{m}(r)$ be ${\left(N-m-1\right)}^{-1}$ times the number of vectors $X_{j}^{m}$ within *r* of $X_{i}^{m}$, and *j* ranges from 1 to *N* − *m* and *j* ≠ *i* to exclude self-matches.

(9) $$C_{i}^{m}(r)=\frac{the\;number\;of\;\{d_{i,j}^{m}\leq r\}}{N-m-1}$$

Define the average similarity

(10) $$C^{m}(r)={\left(N-m\right)}^{-1}\sum_{i=1}^{N-m}C_{i}^{m}(r)$$

Increase the dimension to *m* + 1 and repeat the above steps to calculate $C^{m+1}(r)$. Thus, the statistic of SampEn(m,r) can be defined as follows:

(11) $$SampEn(m,r)=-\underset{N\to \infty }{\lim}\ln(C^{m+1}(r)/C^{m}(r))$$

In fact, the length of sequence is usually finite, so the SampEn can be expressed by the statistic:

(12) $$SampEn(m,r,N)=-\ln(C^{m+1}(r)/C^{m}(r))$$

The SampEn excludes self-matches and only counts the first *N* − *m* vectors [20]. In addition, it represents the degree of self-similarity between two points in the *m*-dimensional phase space after reconstructing the sequence and the probability of generating new patterns when the dimension *m* increases.

### 2.3. Two-Dimensional Entropy

Like in SampEn, given an N-point sequence {u(i):1≤i≤N} and the parameter *m*, form a set of vectors $X_{i}^{m}$ consisting of *m* consecutive *u* values from *i*-th, where *i* ranges from 1 to *N* − *m* + 1. Define $d_{i,j}^{m}$ as the distance between $X_{i}^{m}$ and $X_{j}^{m}$. Next, calculate the similarity of vectors based on the pattern distance and time distance [23] as follows:

(13) $$\mu_{i,j}^{m}=\{\begin{array}{c} \frac{N-m-|i-j|}{N-m}d_{i,j}^{m}\leq r\\ 0\;d_{i,j}^{m}\geq r \end{array}$$

Let $C_{i}^{m}(r)$ be ${\left(N-m-1\right)}^{-1}$ times the sum of similarity, where *j* ranges from 1 to *N* − *m* and *j* ≠ *i*.

(14) $$C_{i}^{m}(r)={\left(N-m-1\right)}^{-1}\sum_{j=1,j\neq i}^{N-m}\mu_{i,j}^{m}$$

Then define $C^{m}(r)$:

(15) $$C^{m}(r)={\left(N-m\right)}^{-1}\sum_{i=1}^{N-m}C_{i}^{m}(r)$$

Similarly, increase the dimension *m* by 1 and get $C^{m+1}(r)$. Considering the probability of generating new information and the degree of pattern self-similarity, TD-entropy is defined as follows:

(16) $$TD-entropy(m,r)=-\underset{N\to \infty }{\lim}\ln(\frac{C^{m+1}(r)}{C^{m}(r)}C^{m+1}(r))$$

which can be evaluated by

(17) $$TD-entropy(m,r,N)=-\ln(\frac{C^{m+1}(r)}{C^{m}(r)}C^{m+1}(r))$$

TD-entropy also excludes self-matches. In contrast, in SampEn, the contributions of all the data points inside the boundary *r* are treated equally, while in TD-entropy the data points inside the boundary *r* are treated differently, where the closer the time distance of vectors is, the higher the similarity.

### 2.4. Fuzzy Entropy

Similarly, Fuzzy entropy excludes self-matches and considers only the first *N* − *m* vectors of length *m* to ensure that $X_{i}^{m}$ and $X_{i}^{m+1}$ are defined for all 1 ≤ *i* ≤ *N* − *m*. For time series {u(i):1≤i≤N}, form vectors as follows:

(18) $$X_{i}^{m}=\{u(i),u(i+1),\ldots ,u(i+m-1)\}-u_{0}(i),\;1\;\leq \;i\;\leq \;N-\;m+\;1$$

where $u_{0}(i)$ is average of sequence consisting of the *m* consecutive *u* values from *i*-th [24].

(19) $$u_{0}(i)=m^{-1}\sum_{j=0}^{m-1}u(i+j)$$

Calculate the distance $d_{i,j}^{m}$ and take the exponential function as the fuzzy membership function. Then, the similarity between two vectors is set as

(20) $$D_{i,j}^{m}=\exp{\left(d_{i,j}^{m}/r\right)}^{n}$$

where *r* is the tolerance of template mismatch, and *n* is the boundary gradient, which is usually recommended to be 2. Let $C_{i}^{m}$ be ${\left(N-m-1\right)}^{-1}$ times the sum of similarity $X_{i}^{m}$ with other vectors $X_{j}^{m}$, where *j* ranges from 1 to *N* − *m* and *j* ≠ *i*.

(21) $$C_{i}^{m}={\left(N-m-1\right)}^{-1}\sum_{j=1,j\neq i}^{N-m}D_{i,j}^{m}$$

Set the average similarity $C^{m}(r)$ and $C^{m+1}(r)$ as follows:

(22) $$C^{m}(r)={\left(N-m\right)}^{-1}\sum_{i=1}^{N-m}C_{i}^{m}(r)$$

(23) $$C^{m+1}(r)={\left(N-m\right)}^{-1}\sum_{i=1}^{N-m}C_{i}^{m+1}(r)$$

The statistic of FuzzyEn(m,r,n) is defined as $\underset{N\to \infty }{\lim}[\ln C^{m}(r)-\ln C^{m+1}(r)]$, which can be replaced by the following statistics:

(24) $$FuzzyEn(m,r,n,N)=\ln C^{m}(r)-\ln C^{m+1}(r)$$

The sample entropy algorithm, defined based on the Heaviside function, applies a hard threshold *r* to measure the similarity, which may cause the instability of the judgement results. FuzzyEn employs the fuzzy membership function, which needs to be equipped with two attributes [26]: (1) continuity and (2) convexity, to measure the similarity of vectors. In this paper, the exponential function was selected as the fuzzy membership function. It can be seen that the FuzzyEn does not directly employ the original sequence but removes a baseline based on it; that is, the FuzzyEn does not depend on the values of the vectors but on the shape.

### 2.5. Sliding t-Test

The sliding *t*-test was developed on the basis of tradition *t*-test, which could only be used to test the significance for a change-point. It is assumed that the sequences divided into double segments due to a mutation point obey the distribution of *F*_1_(*x*) and *F*_2_(*x*). Then, the statistic *T* is defined as follows [34]:

(25) $$T=\frac{{\bar{x}}_{1}-{\bar{x}}_{2}}{\sqrt{\frac{(n_{1}-1)S_{1}^{2}+(n_{2}-1)S_{2}^{2}}{n_{1}+n_{2}-2}(\frac{1}{n_{1}}+\frac{1}{n_{2}})}}$$

where ${\bar{x}}_{i}$ and $S_{i}^{2}$ denote the mean and variance, respectively, of the sample sequences, and *n*_1_ and *n*_2_ are the amount of samples. The statistic *T* obeys a *t* distribution with a degree of freedom (*n*_1_ + *n*_2_ − 2). Taking a confidence level α, use a two-tailed test to verify the hypothesis *F*_1_(*x*) = *F*_2_(*x*). When the statistic |*T*| exceeds *t_α_*_/2_, there is a significant difference between *F*_1_(*x*) and *F*_2_(*x*); thus, the original assumption is not accepted. When the statistic |*T*| is less than *t_α/_*_2_, the difference is not significant, and this assumption can be accepted. In addition, if there are multiple points that make |*T*| > *t_α/_*_2_, the point at which |*T*| reaches the maximum is selected as the most likely variation point. (The year corresponding to the peak or trough of the wave is the year of the mutation).

### 2.6. Brown–Forsythe Test

In statistics, analysis of variance is a method of analysing the difference in the mean level of experimental data, and its main purpose is to quantitatively distinguish the difference between the experimental results caused by different observation conditions and the experimental results caused by random factors to determine whether there are systemic factors that play a role in the experiment. The statistics are as follows [35]:

(26) $$F=\sum_{i=1}^{m}n_{i}{\left({\bar{x}}_{i}-\bar{x}\right)}^{2}/\sum_{i=1}^{m}\left(1-n_{i}/N\right)S_{i}^{2}$$

(27) $$f=1/\sum_{i=1}^{m}c_{i}^{2}/\left(n_{i}-1\right)$$

(28) $$c_{i}=(1-n_{i}/N)S_{i}^{2}/\left[\sum_{i=1}^{m}\left(1-n_{i}/N\right)S_{i}^{2}\right]$$

The statistic obeys the *F* distribution with a degree of freedom (*m* − 1, *f*), where *m* is the number of groups, $n_{i}$ is the number of samples in the *i*-th group, *N* is the total number of samples, ${\bar{x}}_{i}$ is the sample mean of the *i*-th group, $\bar{x}$ is the mean of the total sample, and $S_{i}^{2}$ is the variance of the *i*-th group.

### 2.7. Cumulative Anomaly

Cumulative anomaly is a test method based on the mean value. By observing the curve of cumulative deviation from the mean, the trend and the variability in the streamflow can be detected. For the time series $\left\{X_{i}:1\leq i\leq n\right\}$, the cumulative anomaly at a certain moment is expressed as follows [36]:

(29) $${\hat{x}}_{t}=\sum_{i=1}^{t}\left(x_{i}-\bar{x}\right)$$

where *t* ranges from 1 to *n*, and $\bar{x}$ is the average of sequence. The curve shows an upward trend, indicating a positive anomaly; a decreasing trend indicates a negative anomaly.

## 3. Application

### 3.1. Gauging Location

In this study, the multiple entropies are carried out with application to the four gauging stations on the mainstream of the Weihe River with a drainage area of approximately 135,000 km^2^. The Weihe River, the largest tributary of the Yellow River, extends from western Weiyuan County in Gansu Province more than 800 km east to Tongguan County in Shaanxi Province and finally flows into the Yellow River [37,38].

**Figure 1:**
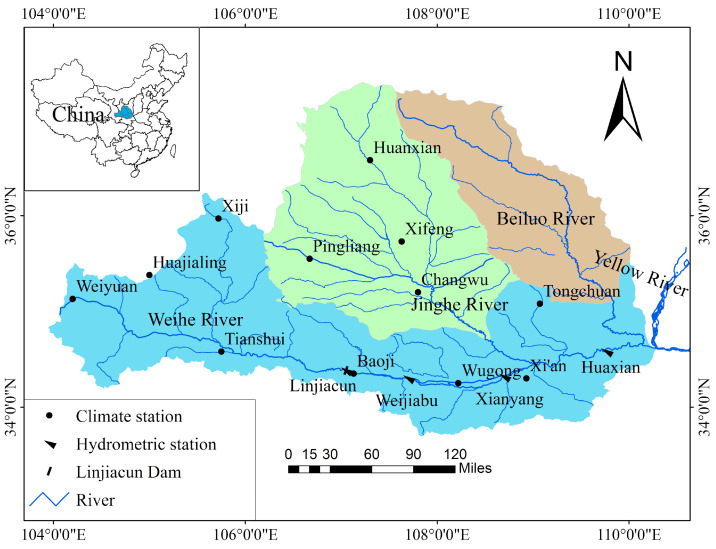
Geographical location of Mainstream Area of the Weihe River basin in China and spatial distribution of the hydrometric stations and the climate stations used in this study.

The geographical locations of the four gauging stations adopted in this study, Linjiacun station, Weijiabu station, Xianyang station and Huaxian station, are depicted in Figure 1. Among them, Linjiacun station is the upstream control station, and Huaxian station is the downstream control station for the Weihe River flowing into the Yellow River. The spatial distributions of the four hydrometric stations and twelve climate stations are shown in Figure 1.

### 3.2. Data Used

The monthly discharge data from the four basic gauging stations in the Weihe River basin over 1954–2016 (total of 756 months) were obtained from the Hydrological Yearbooks compiled and published by the Ministry of Water Resources of China. The monthly average streamflow time series were utilized in this study to interpret the spatial difference and variation in streamflow complexity based on the multiple entropies. Basic information of the gauging stations and descriptive statistics of the data are summarized in Table 1, including, for each station, maximum, minimum, standard deviation (SD) and coefficient of variation (Cv).

It can be seen from Table 1 that the differences between the maximum and the mean were in the range of approximately 7–10 standard deviations, strongly positively skewed. The Cv value of monthly runoff is relatively large at over 0.7, indicating a drastic temporal variability for the monthly discharge.

**Figure 2:**
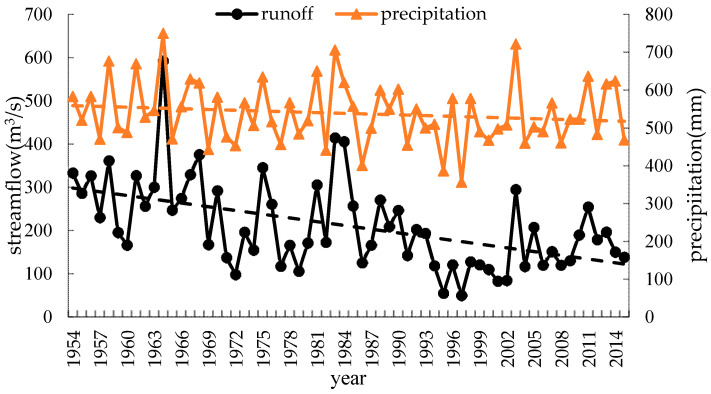
Changes and overall trend of annual runoff and annual precipitation in the Weihe River basin. The runoff data used is the measured monthly discharge record of Huaxian station.

The whole watershed is influenced by an arid and semi-arid climate, and the monthly fluctuation in *P* is obvious since most of the basin is located within the East Asian monsoon climatic belt. The precipitation data of 12 climate stations from China Meteorological data network are processed to obtain the watershed areal rainfall. The average annual precipitation in the basin is approximately 545.0 mm, and the Cv value of precipitation is 0.17–0.19. The dynamic changes and the evolution tendency of the annual runoff and annual precipitation in the Weihe River basin are shown in Figure 2, which reflects that rainfall and runoff exhibit an approximately consistent process of change, and both of them appear to have a certain downward trend, especially for runoff. Obviously, the change and variability in runoff are more complicated and significant than those of precipitation. The reduction in rainfall is a factor in the reduction in runoff; furthermore, anthropogenic effects such as land use and land cover change, and diversion irrigation in the basin, which can introduce additional randomness into streamflow, may be the main cause of the decline and dramatic changes in runoff.

### 3.3. Parameter Selection

The parameters involved in ApEn, SampEn, TD-entropy and FuzzyEn are *r*, *m* and *N*. The *r* is a given threshold, that is, the tolerance for pattern mismatch. For a small value of *r*, the excessive interferential information would be counted. In contrast, some details would be neglected. The parameter *m* is the embedded dimension. The parameter *N* represents the total length of the sequence in calculating the static entropy value, while it represents the size of the sliding window in calculating the entropy value in the form of a sliding window. According to the works performed by other researchers [12,17,20], it was recommended that *r* takes the value of (0.1–0.25) times SD (the standard deviation of the original sequence) and *m* takes the value 2. Based on several calculations, when the parameter *r* was set as 0.15SD and *m* was set as 2, relatively stable results can be obtained.

For FuzzyEn, there is one more parameter *n*, the boundary gradient, and *n* = 2 in this paper.

### 3.4. Spatial Difference for Streamflow Complexity

To investigate the spatial distribution of streamflow complexity in the Weihe River basin, the static entropy values of the monthly average streamflow time series over 1954–2016 (with 756 samples) of each gauging station are calculated first. The results are shown in Table 2.

Weijiabu station obtains the minimum values for all four entropies, and Huaxian has the maximum entropies values. Therefore, a systematic increase in the entropies values exists along the Weihe River, except for one site, Linjiacun station. According to the nature of entropy, the larger the entropy value is, the higher the complexity of the system. It shows that there is a spatial difference in the streamflow complexity along the Weihe River; that is, the complexity of the streamflow time series increases along the Weihe River, except for the Linjiacun station, and the Huaxian station has the highest complexity, and the Weijiabu station has the smallest complexity. There are many factors affecting the streamflow, and the causes of the complexity difference of streamflow on the space may be related to the catchment area, inflow composition and human activities. The complexity increases gradually from the Weijiabu station to the Huaxian station since the downstream flow not only inherits the incoming water from upstream but also accepts the inflow from the tributaries, and at the same time, human activities are more frequent. That is, the input and output relationship of the streamflow is more complicated downstream, and the complexity is higher. Moreover, Linjiacun station is the exit station of the upstream Linjiacun reservoir. Affected by the reservoir operation, the natural state of the streamflow is broken, and the measured streamflow time series has higher complexity.

### 3.5. Dynamic Change for Streamflow Complexity

**Figure 3:**
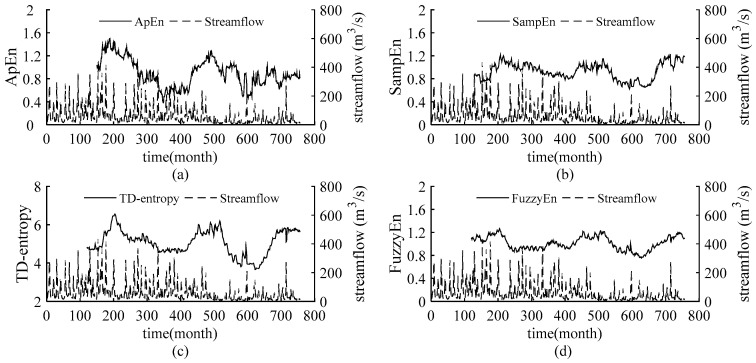
The relation curves between the measured monthly streamflow and the entropies for the Linjiacun gauging station. (**a**) The sliding ApEn curve of measured streamflow; (**b**) the sliding SampEn curve of measured streamflow; (**c**) the sliding TD-entropy curve of measured streamflow; (**d**) the sliding FuzzyEn curve of measured streamflow.

**Figure 4:**
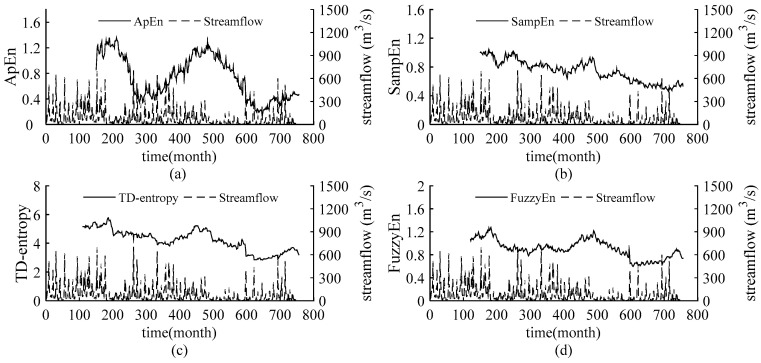
The relation curves between the measured monthly streamflow and the entropies for the Weijiabu gauging station. (**a**) The sliding ApEn curve of measured streamflow; (**b**) the sliding SampEn curve of measured streamflow; (**c**) the sliding TD-entropy curve of measured streamflow; (**d**) the sliding FuzzyEn curve of measured streamflow.

**Figure 5:**
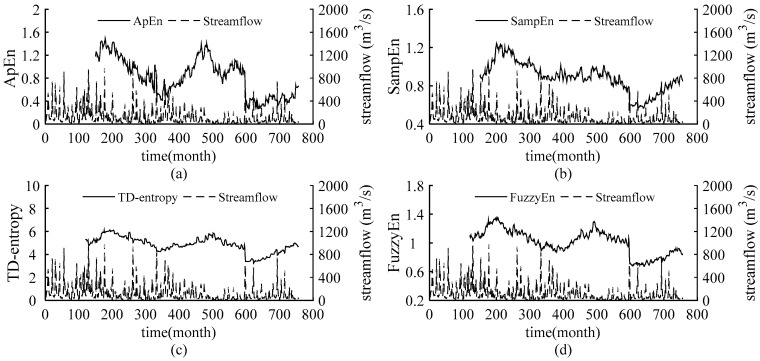
The relation curves between the measured monthly streamflow and the entropies for the Xianyang gauging station. (**a**) The sliding ApEn curve of measured streamflow; (**b**) the sliding SampEn curve of measured streamflow; (**c**) the sliding TD-entropy curve of measured streamflow; (**d**) the sliding FuzzyEn curve of measured streamflow.

**Figure 6:**
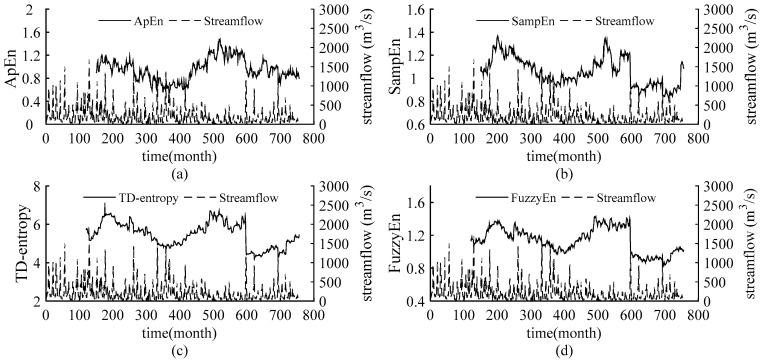
The relation curves between the measured monthly streamflow and the entropies for the Huaxian gauging station. (**a**) The sliding ApEn curve of measured streamflow; (**b**) the sliding SampEn curve of measured streamflow; (**c**) the sliding TD-entropy curve of measured streamflow; (**d**) the sliding FuzzyEn curve of measured streamflow.

The static entropy values obtained above reflect the overall complexity of the streamflow in the Weihe River and cannot quantify the dynamic change in streamflow complexity over time. Introducing sliding skill into the static entropies, sliding ApEn, sliding SampEn, sliding TD-entropy and sliding FuzzyEn are employed to further depict the dynamic characteristic of the complexity of streamflow time series. Set a sliding window of size *N* and move along the sequence until the end of the sequence. The entropy values are calculated for the data in each window and sequentially are connected to obtain the dynamic curves of the four entropies of the streamflow sequences. We increased the window length from 60 to 200 months, with one month as the sliding step, to verify the results under different windows, so as to determine the window size for obtaining stable results. Finally, the window size of sliding ApEn and sliding SampEn is 150 months and that of sliding TD-entropy and sliding FuzzyEn is 110 months. The entropy curves are compared with the runoff process, and the results are shown in Figure 3, Figure 4, Figure 5 and Figure 6.

As shown in Figure 3, Figure 4, Figure 5 and Figure 6, throughout the 63-year study period, the entropy curves of ApEn, SampEn, TD-entropy and FuzzyEn of the monthly average streamflow for each station show a similar tendency of evolution and fluctuate sharply with obvious phase characteristics. There is an approximate inverse relation between the runoff process and the corresponding entropy values for each station, that is, the runoff increases, the corresponding entropy value decreases; contrarily, the runoff decreases, and the corresponding entropy value would increase.

Figure 3 shows the dynamic change of the monthly streamflow complexity and the corresponding monthly streamflow process in Linjiacun station. A similar behaviour was observed for ApEn, SampEn, TD-entropy and FuzzyEn, having values in the intervals (0.3842; 1.5021), (0.623; 1.2233), (3.6617; 6.5556), and (0.755; 1.2657), respectively, where the values of TD-entropy are relatively high, and it displays the largest range of entropy values. The curves of TD-entropy and FuzzyEn are smoother than those of ApEn and SampEn and can depict the characteristics between different dynamic structures. Then, it can be clearly seen from the Figure 3 that there is obvious peak-to-valley correspondence between the entropy curves and the runoff process. The entropy curves reach the peak around the 205th month, but the runoff shows a valley value; after that, the entropy curves begin to decline and remain stable for a long time. In the following 475th month, the entropy curves show a near peak, and runoff appears a valley value. Before the peak of runoff in the 600th month, the entropy curves have a peak; when the runoff reaches its peak, the entropy values drop sharply; in the 715th month, a large peak of the runoff leads to a temporary valley in the entropy curves. Due to the complexity of data with different dynamic properties being discrepant, the entropy value remains relatively stable when the dynamic structure is unchanged, and yet the entropy value changes significantly when the system dynamic structure shifts. At the abovementioned positions, the 205th, 475th and 600th month, the evolution trend in the complexity changed, indicating that the dynamic structure of the sequence has been changed and that the streamflow may have abrupt changes. For the 715th month, though the entropy curves have a brief drop, the evolution trend remains unchanged.

The change in streamflow process and corresponding entropy values for the Weijiabu station is shown in Figure 4. The ApEn and SampEn curves differ obviously, while the TD-entropy and FuzzyEn have the same trends. TD-entropy is depicted as the largest range of entropy values in the intervals (2.8331; 5.7735), which can better describe the small changes in runoff complexity, and SampEn has the smallest range of entropy values, ranging in the intervals (0.4634; 1.0356). The runoff presents obvious phase characteristics. As shown in Figure 4c,d, the runoff shows two significant reductions and a significant increase, respectively at the 180th month, the 475th month and the 600th month, yet the entropy curves exhibit two peaks and one sharp drop. However, the curves in Figure 4a,b, show no obvious characteristics near the 600th month. Compared with the Linjiacun station, there is no evident uptrend in the complexity of the streamflow sequence after the 600th month.

By analyzing Figure 5 and Figure 6, we also find the same peak-to-valley correspondence. The evolution process of the streamflow complexity for the Xianyang station and the Huaxian station are rough similar. Similar to the Linjiacun station, both show two peaks and one drop, respectively, at approximately the 205th, 475th and 600th months. In addition, TD-entropy revealed the largest range of entropy values among the four entropies. Figure 5 and Figure 6 show that the curves of TD-entropy and FuzzyEn possess similar behaviours, and both of them are smoother than the curves of the other two.

Precipitation is the most direct factor affecting runoff, and the changes in streamflow should be related to changes in precipitation. Figure 2 in this paper and the literature [39,40] show that the interannual change in runoff is greater than that of precipitation in the Weihe River mainstream. Therefore, anthropogenic influences on the variation in complexity of the runoff series in the basin should be considered. Since the 1970s, large-scale water conservancy construction and terrace development began in the Weihe River basin [41], accompanied by changes in underlying conditions and confluence conditions, and a decline in runoff efficiency. Thus, the evolution trend of streamflow changed around 1970.The cultivated-land, woodland and grassland are main usage in the region, accounting for about 95% [42] of the area. From 1980 to 2000, intense human activity changed land use and land cover change (LUCC) of the region, with increased cultivated-land and reduced woodland and grassland [42,43], and the LUCC in 1980–1990 transformed significantly than that in 1990–2000 [42]. Also, the Normalized Difference Vegetation Index (NDVI) show a non-significant uptrend in this period [44]. It can be seen form the Figure 3, Figure 4, Figure 5 and Figure 6 that the runoff complexity of the same period increases first and keeps high values. Moreover, in 1990s, the precipitation in the basin decreased, coupled with the increase in water withdrawal outside the river. The combined effect of multiple factors resulted in the reduction in streamflow and the occurrence of short-duration drought in this period. The ordered state of the streamflow changed again, and the complexity of the system increased. Compared with the period of 1980-1999, the land use change, from 2000 to 2015, is significant with a decrease in farmland and an increase in forest and grassland benefitting from the implement of Grain-to-Green [43,45], and the increase in the precipitation corresponds to the increase trend of NDVI after 2000 [44]. In 2003, a rainstorm occurred in the Weihe River basin, suddenly increasing the streamflow. It can be seen from the Figure 4, Figure 5 and Figure 6 that the entropy curves drop sharply and the trend of entropy change, indicating the ordered state of the streamflow time series was broken. The effects of climate change and human activities on the complexity of the streamflow in the Weihe River basin can be reflected in the dynamic changes in the ApEn, SampEn, TD-entropy and FuzzyEn curves.

Detection of the changes in the entropies values and curves and analysis of the anthropogenic activities in the Weihe River basin strengthened the conclusions that the change-points of streamflow may have occurred around the 205th month (in 1971), 475th month (in 1993) and 600th month (in 2003) for the Linjiacun station, Xianyang station and Huaxian station, and change-points may have occurred around the 180th month (in 1968), 475th month (in 1993) and 600th month (in 2003) for the Weijiabu station.

In addition, it can be seen from Figure 3, Figure 4, Figure 5 and Figure 6 that the local entropy values in the TD-entropy and FuzzyEn curves fluctuate less, indicating that the two entropies are not sensitive to interference information. However, the entropy values change sharply when the system structure changes. Therefore, the dynamic structural change characteristics of the sequences can be effectively and comprehensively displayed by the two methods. Additionally, the TD-entropy values range from 2.5 to 7 and should be obviously larger than ApEn and SampEn, indicating that TD-entropy can better diagnose small changes in the complexity of the hydrological system.

### 3.6. Comparative Analysis of Streamflow Variability

**Figure 7:**
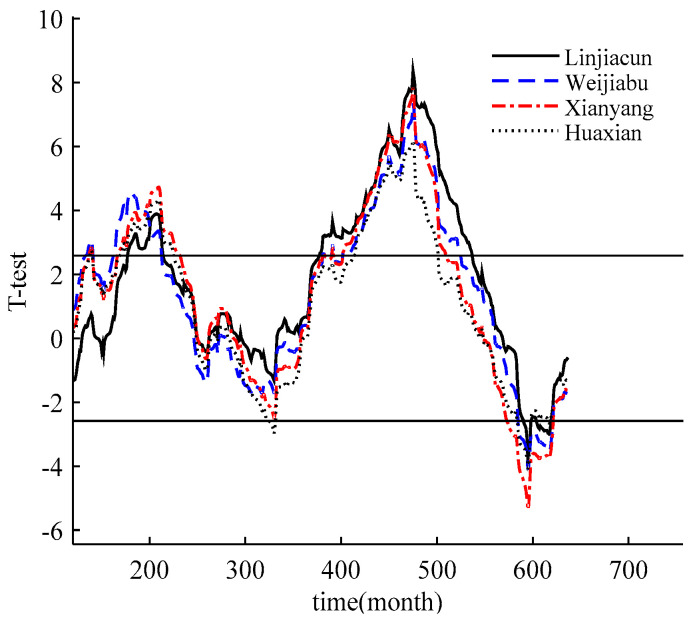
Sliding *t*-test curves of monthly mean streamflow series.

The sliding *t*-test is used to verify the results of the sliding entropy methods. Taking the significance level *α* = 0.01 (*T*_α/2_ = 2.584), the two-tailed test is selected to verify whether the hypothesis *F*_1_(*x*) = *F*_2_(*x*) held. It can be seen from both Figure 7 and Table 3 that the statistic at the peaks or valleys exceeds the significance level. At the 180th, 475th and 595th months for the Weijiabu station, the *T* statistic reaches the peak or trough, and the absolute values of the statistic are much greater than *T*_α/2_, indicating that there are significant differences between the sequences before and after the possible change points. In addition, *T*_180_ and *T*_475_ (*T*_180_ represents the statistical value at the 180th month) are both positive values, indicating that the sequence had a mutation of the average decrease. However, *T*_595_ is a negative value, indicating that the sequence has a jump in the average increase. The absolute values of the statistics of the Linjiacun station, Xianyang station and Huaxian station in the 209th, 475th and 595th months are all greater than *T*_α/2_. Similarly, the sequence has two change points with an average decrease and one variation with an average increase.

The results of the Brown–Forsythe test are shown in Table 4. Using the method of segmenting the sample sequence, a sequence containing only one possible variation point is divided into two segments, and then whether there are significant differences between the two sample sequences is verified. The statistical values obtained are significantly higher than the significance level of 95% (Table 4), indicating that there was a significant difference between the sample sequences before and after the above possible variation points.

**Figure 8:**
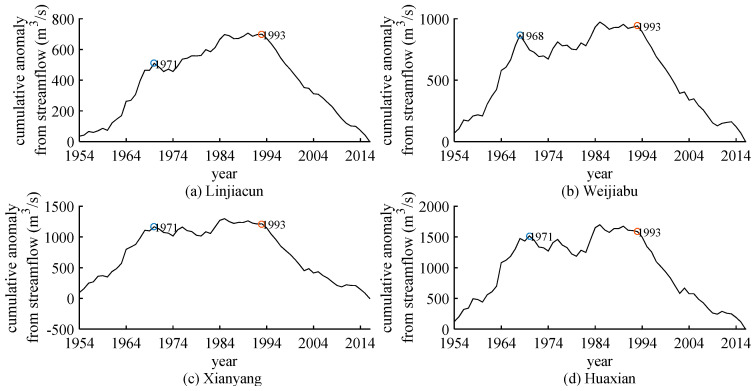
Cumulative anomaly analysis of annual streamflow for four gauging stations. (**a**) Cumulative deviation curve of runoff from mean value in Linjiacun station; (**b**) cumulative deviation curve of runoff from mean value in Weijiabu station; (**c**) cumulative deviation curve of runoff from mean value in Xianyang station; (**d**) cumulative deviation curve of runoff from mean value in Huaixan station.

Figure 8 shows the anomaly accumulation curves of annual runoff for four hydrological stations. The cumulative anomaly curve of annual runoff for Linjiacun station, shown in Figure 8a, increase significantly from 1954 to 1971, decline slightly after 1971, and then increase but non-significant than before, and there is a significant decline in runoff after 1993. The streamflow shows the variability around 1971 and 1993. The cumulative anomaly curve for Weijiabu station is shown in Figure 8b. The curve exhibits two change-points in 1968 and 1993, respectively, presenting a conspicuous uptrend in 1954–1968 and a sharply downtrend after 1993. Moreover, the cumulative anomaly curves of Xianyang station and Huaixan station also demonstrate the variability in runoff time series around 1971 and 1993, as in Linjiacun station. In addition, it can be found that there is no evident change characteristic in 2003 for four gauge stations. The possible reason is that the runoff was relatively dry in 1990s, despite increasing after 2003 but still less than the mean runoff.

Table 5 shows the comparison results of the variation points obtained by different methods. By comparison, it can be found that there are slight differences in the months of possible change points obtained employing different methods, but the corresponding years are consistent. In addition, entropies can detect all the variation points compared with cumulative anomaly. The quantitative detection method has good effect in variability detection, and the qualitative analysis methods may not be able to detect all variation points such as cumulative anomaly. This confirms that ApEn, SampEn, TD-entropy and FuzzyEn can effectively characterize the complexity of the streamflow sequences and identify their variation. That is, the variation years of the runoff series are in 1968, 1993 and 2003 for the Weijiabu station, and the variation years of the runoff series are 1971, 1993 and 2003 for the Linjiacun station, Xianyang station and Huaxian station.

## 4. Conclusions

In this paper, ApEn, SampEn, TD-entropy and FuzzyEn were employed to analyse the complexity of the observed monthly streamflow of four gauging stations in the Weihe River basin. The following conclusions can be drawn from this study:

- ApEn, SampEn, TD-entropy and FuzzyEn are effective in the quantitative analysis of the complexity of hydrologic systems and can effectively characterize the variation in streamflow complexity. It has been confirmed that ApEn and SampEn detected streamflow complexity in this paper. In addition, the satisfactory results lead us to conclude that TD-entropy and FuzzyEn have better continuity and relative consistency and that TD-entropy has a larger range of entropy values. These two entropies are more suitable for short and noisy hydrologic time series, more effectively identifying the streamflow complexity.
- The streamflow complexity undergoes spatial difference across the Weihe River basin. The complexity of the streamflow increases gradually along the Weihe River, except for the Linjiacun station, whose complexity increases due to the reservoir operation.
- The dynamic changes in the complexity of the streamflow series have been surveyed for four gauging stations, employing the four sliding entropies. It was found that there is a relation of peak-to-valley correspondence between the runoff process and the entropy values for each station, and, the observed streamflow series at the Weijiabu station changed in 1968, 1993 and 2003 and changed in 1971, 1993 and 2003 at the Linjiacun station, Xianyang station and Huaxian station. The sliding *t*-test and Brown–Forsythe test confirmed the results of the entropies.
- This paper introduces TD-entropy and FuzzyEn into the hydrologic system and expects to provide more effective methods for analysing the complexity of hydrologic systems. The results could be very useful in identifying variation points of streamflow series, a key in modelling and prediction.

## Figures and Tables

**Table 1:** Basic information of monthly streamflow data for selected gauging stations.

Station	Longitude	Latitude	Control Area (km^2^)	Max (m^3^/s)	Min (m^3^/s)	Mean (m^3^/s)	SD (m^3^/s)	Cv
Linjiacun	107°03′ E	34°23′ N	30,661	470	0.4	62.17	6.91	0.95
Weijiabu	107°42′ E	34°18′ N	37,012	705	0	86.9	9.24	0.77
Xianyang	108°42′ E	34°19′ N	46,827	974	2.32	122.88	8.35	0.83
Huaxian	109°46′ E	34°25′ N	106,498	1690	2.7	208.66	8.16	0.87

**Table 2:** The static entropy values of streamflow time series for four gauging stations.

Stations	ApEn	SampEn	TD-Entropy	FuzzyEn
Linjiacun	1.1056	0.7815	4.4371	1.0061
Weijiabu	0.9408	0.6188	3.7067	0.9186
Xianyang	1.0248	0.7819	4.3723	0.9972
Huaxian	1.1106	0.9796	5.0415	1.1855

**Table 3:** The corresponding values of statistic *T* for peaks and troughs.

Stations	*T*_180_	*T*_209_	*T*_475_	*T*_595_
Linjiacun	-	3.8947	8.3615	−3.7514
Weijiabu	4.568	-	7.3648	−4.1015
Xianyang	-	4.7389	7.8312	−5.3698
Huaxian	-	4.3382	6.1971	−4.0848

**Table 4:** Statistic F of monthly mean streamflow series for four gauging stations.

Stations	*F*_180_	*F*_202_	*F*_480_	*F*_595_
Linjiacun	-	7.0307	93.8275	20.7573
Weijiabu	16.966	-	67.8543	21.4195
Xianyang	-	15.2442	68.8939	32.6696
Huaxian	-	10.2332	38.499	18.5833

*F*_180_ indicates that the statistic *F* reaches the maximum at 180th month.

**Table 5:** Comparison of the results for the complexity variability obtained by different methods.

Stations	Entropy Methods	*t*-Test	Brown–Forsythe	Cumulative Anomaly	Variation Points
	(Month)	(Month)	(Month)	(Year)	(Year)
Linjiacun	205, 475, 600	209, 475, 595	202, 480, 595	1971, 1993	1971, 1993, 2003
Weijiabu	180, 475, 600	180, 475, 595	180, 480, 595	1968, 1993	1968, 1993, 2003
Xianyang	205, 475, 600	209, 475, 595	202, 480, 595	1971, 1993	1971, 1993, 2003
Huaxian	205, 475, 600	209, 475, 595	202, 480, 595	1971, 1993	1971, 1993, 2003
